# Sensitive Simultaneous Detection of Seven Sexually Transmitted Agents in Semen by Multiplex-PCR and of HPV by Single PCR

**DOI:** 10.1371/journal.pone.0098862

**Published:** 2014-06-12

**Authors:** Fabrícia Gimenes, Fabiana Soares Medina, André Luelsdorf Pimenta de Abreu, Mary Mayumi Taguti Irie, Isis Baroni Esquiçati, Natália Malagutti, Vinícius Rodrigo Bulla Vasconcellos, Michele Garcia Discacciati, Marcelo Gialluisi Bonini, Silvya Stuchi Maria-Engler, Marcia Edilaine Lopes Consolaro

**Affiliations:** 1 Section of Clinical Cytology, Department of Clinical Analysis and Biomedicine, State University of Maringá, Maringá, Paraná, Brazil; 2 Clinical Chemistry and Toxicology Department, School of Pharmaceutical Sciences, University of São Paulo, São Paulo, Brazil; 3 College of Medicine, Department of Pharmacology, University of Illinois at Chicago, Illinois, Chicago, United States of America; Rush University, United States of America

## Abstract

Sexually transmitted diseases (STDs) may impair sperm parameters and functions thereby promoting male infertility. To date limited molecular studies were conducted to evaluate the frequency and type of such infections in semen Thus, we aimed at conceiving and validating a multiplex PCR (M-PCR) assay for the simultaneous detection of the following STD pathogens in semen: *Chlamydia trachomatis*, *Neisseria gonorrhoeae*, *Mycoplasma genitalium*, *Trichomonas vaginalis*, Herpes virus simplex (HSV) −1 and −2, and *Treponema pallidum*; We also investigated the potential usefulness of this M-PCR assay in screening programs for semen pathogens. In addition, we aimed: to detect human *Papillomavirus* (HPV) and genotypes by single PCR (sPCR) in the same semen samples; to determine the prevalence of the seven STDs, HPV and co-infections; to assess the possibility that these infections affect semen parameters and thus fertility. The overall validation parameters of M-PCR were extremely high including agreement (99.2%), sensitivity (100.00%), specificity (99.70%), positive (96.40%) and negative predictive values (100.00%) and accuracy (99.80%). The prevalence of STDs was very high (55.3%). Furthermore, associations were observed between STDs and changes in semen parameters, highlighting the importance of STD detection in semen. Thus, this M-PCR assay has great potential for application in semen screening programs for pathogens in infertility and STD clinics and in sperm banks.

## Introduction

It is estimated that over 340 million new cases of sexually transmitted diseases (STDs) occur annually throughout the world, with the highest incidence in developing countries. Pelvic inflammatory disease, infertility, ectopic pregnancy, chronic pelvic pain, neonatal morbidity and mortality, and genital cancer all have been associated to some degree to STDs [1]. Furthermore, an increasing risk of acquiring these infections may have a substantial impact on global susceptibility to HIV-1 transmission [2]–[4].

Since 1993, the World Health Organization (WHO) has established a role for genital tract infections in human infertility [5]. Most male STD pathogens seem to be involved in male reproductive tract problems, including genital injury, infections of semen, prostatitis, urethritis, epididymitis and orchitis [6]. There is possible involvement in female infertility, pregnancy complications and transmission from infected mothers to the fetus or newborn [7]. According to recent findings 15–20% of infertile male subjects are affected by semen infection [8], and most data have been concordant with respect to the relevance of STDs to male infertility [7], [8]. Several STDs in semen were associated with poor sperm quality [9] and decreased sperm concentration and motility [7]. However, there are few studies evaluating these aspects, and additional epidemiological studies in different populations and clinical scenarios are needed to determine the real impact of STD pathogens on male infertility.

Semen STDs are prevalent in asymptomatic males over extended periods of time [7]. Chronic or inadequately treated infections play a greater role in infertility than acute infections, although in many cases the exact etiological agents are unknown [1]. An undiagnosed infection can have potentially significant implications for individual's and population health [10]. For this reason, some authors have proposed the implementation of screening programs for semen STD pathogens in infertility and STD clinics and in sperm banks [11], [12].

Over the past three decades, diagnostics for STDs have depended nearly exclusively on traditional methods, such as culture, enzyme immunoassay, and fluorescent antibody staining, especially in developing word [13]. The need for rapid, sensitive, versatile and low cost diagnostic tools for the screening of semen samples is evident.

Here we report a validated multiplex polymerase chain reaction (M-PCR) diagnostic method to simultaneous screen for *Chlamydia trachomatis*, *Neisseria gonorrhoeae*, *Mycoplasma genitalium*, *Trichomonas vaginalis*, herpes virus (HSV) types 1 and 2, and *Treponema pallidum* in semen. Human *Papillomavirus* (HPV) and genotypes also were detected by single PCR (sPCR) in the same semen samples. We anticipate that M-PCR will potentially impact diagnostics of STD in semen and contribute to diminish male infertility in the near future.

## Materials and Methods

### Study population and semen specimens

The study population was selected in the Sperm Analysis Section (SAS) of Clinical and Research Laboratory of State University of Maringá (UEM)/Brazil between January 2012 and June 2013. Semen analysis was requested as part of a work-up for conjugal infertility investigations after failing to conceive after one year of unprotected intercourse or as a post-vasectomy sterility control. Men who had reproductive system abnormalities (e.g., varicocele), symptoms of genitourinary infections, or those that had received antibiotics within the preceding 3 months or infertility therapy within the preceding year were excluded from the study. Considering this exclusion criteria, the semen samples analyzed between January 2011 and June 2012 (prior to the study period) by SAS/UEM, the sample size calculated for our study was fixed at least 72 semen samples (confidence interval of 95% and an error estimate of 5%; EPI INFO 7.0 software).

A total of 76 men were included in the present study with a mean age of 33.4 ± 7.2 years (range 19–51). The men signed a consent form, and this study was approved by the Committee for Ethics in Research Involving Humans at the State University of Maringá (UEM)/Paraná, Brazil (No. 162.209/2012).

Prior to semen collection and analysis, the men were asked to abstain from sexual intercourse or masturbation for 3–5 days. All samples for analysis were collected on site and into standard containers that had previously been shown not to have any cytotoxic effects on human spermatozoa [15]. Immediately after semen collection, the samples were placed in an incubator and liquefied at 37°C for up to 30 minutes. Semen analysis was performed according to the World Health Organization [15] criteria to determine the following variables: seminal volume, pH, sperm concentration, vitality, total progressive motility (category [a + b]), rapid progressive motility (category [a]) and morphology (normal forms) [15]. Oligospermia was defined as sperm concentration <20×10^6^/ml, asthenospermia as sperm motility <50% (category [a + b]), necrospermia as sperm vitality <50% and teratospermia as normal morphology <30%.

### Genomic DNA extraction

DNA was extracted using an AxyPrep Body Fluid Viral DNA/RNA Miniprep Kit (Axygen, CA, USA) according to the manufacturer's instructions. The quality and quantity of purified DNA were measured by spectrophotometry (NanoDrop 2000 Spectrophotometer, Thermo Scientific, Wilmington, USA).

### Multiplex-PCR for 7 STD pathogens in semen

We made selected adaptations to previously designed M-PCR assays to achieve simultaneous detection of the seven selected STDs [13], [14], [16]. Primers were characterized by compatible melting temperatures and yielded amplicons with sizes easily separable by agarose gel electrophoresis (Table 1). The optimized protocol consisted of a reaction mixture of 25 µL containing 2.5 mM of each dNTP, 0.6 mM of MgCl_2_, 25 mM of each primer, 5 µL of extracted DNA (50 ng of total sample) and 1 U of Platinum Taq DNA polymerase (Invitrogen, CA, USA). The PCR conditions comprised thirty-five amplification cycles of denaturation for 10 minutes at 94°C, annealing for 1 minute at 62°C, extension for 1 minute at 72°C and final extension for 10 minutes at 72°C (Thermal cycler, Biosystem, CA, USA). The M-PCR products were electrophoresed on a 2.5% agarose gel stained with 1 µg/mL ethidium bromide. In a few cases in which the bands have not been viewed in the 2.5% agarose gel, we analyzed products using an 8% polyacrylamide gel. Positive controls for all studied STDs were derived from positive clinical samples detected by reference methods, including culture and/or single-target PCR (sPCR). For validation, sPCR was also performed for the seven microorganisms in all samples studied and for positive controls using the same primers as for the M-PCR. sPCR (gold standard) is generally more sensitive than M-PCR, and cross-reactivity, which can occur during M-PCR, is avoided [17].

**Table 1:** Oligonucleotide primers used in the M–PCR assay.

Pathogens	Primers	Oligonucleotides (5′– 3′)	Amplicon size (bp)
CT	Forward Reverse	TCTTTTTAAACCTCCGGAACCCACTT GGATGGCATCGCATAGCATTCTTTG	361
TP	Forward Reverse	GGAGAAGTTTCACTTCGTGGA CTCGCGTCATCACCGTAGTA	291
HSV-2	Forward Reverse	CATGGGGCGTTTGACCTC TACACAGTGATCGGGATGCT	249
MG	Forward Reverse	ACCTTGATGGTCAGCAAAACTT CCTTTGATCTCATTCCAATCAGTA	193
TV	Forward Reverse	CCAGAAGTGGGCTACACACC ATACCAAGGCCGGAAGCAC	170
NG	Forward Reverse	CGGCAGCATTCAATTTGTT AAAAAGCCGCCATTTTTGTA	162
HSV-1	Forward Reverse	CTGTGGTGTTTTTGGCATCA GGTTGTGGAGGAGACGTTG	123

M–PCR, multiplex–PCR; CT, *C. trachomatis*; TP, *T. pallidum*; HSV–1/–2: herpes virus simplex; MG, *M. genitalium*; TV, *T. vaginalis*; NG, *N. gonorrhea*; bp, base pairs.

All clinical samples were also tested using human β-globin-specific primers GH20/PC04 as an internal control for amplification and DNA integrity under the same conditions as the M-PCR or sPCR reactions.

### sPCR for HPV detection

This method has been in use in our laboratory for several years and consists of HPV-PCR amplification carried out using primers MY09 (5'CGTCCMAARGGAWACTGATC-3′) and MY11 (5′-GCMCAGGGWCATAAYAATGG-3′) as described previously [18]. The reaction consisted of 2.5 mM of each dNTP, 1 U of Taq DNA polymerase (Invitrogen, Carlsbad, CA), 0.6 mM of MgCl_2_, 25 mM of each primer and 50 ng of extracted DNA for a final volume of 15 µL. PCR products were electrophoresed on a 1.0% agarose gel, stained with 1 µg/mL ethidium bromide, and photodocumented under UV light. The samples that gave a positive result by PCR were further analyzed by HPV genotyping. Co-amplification of the human β-globin gene was performed as an internal control using primers GH20 (5′-GAAGAGCCAAGGACAGGTAC-3′) and PC04 (5′-CAACTTCATCCACGTTCACC-3′) under the same conditions as the HPV-PCR. Two types of controls were also included in each reaction series: ‘no-DNA’ (negative control) and ‘HPV-positive DNA’ (positive control).

### PCR-RFLP for HPV genotyping

HPV-positive samples were genotyped using a PCR-RFLP (Restriction Fragment Length Polymorphism) as described previously [19]. Ten microliters of each PCR sample were digested in a final volume of 15 µL with the restriction enzyme *Hpy*CH4 V (New England Biolabs, Ipswich, MA, USA) according to the manufacturer's instructions. Restriction fragments were resolved on 8% polyacrylamide gels. HPV genotypes were determined by analyzing each band with Labimage 1D software (Loccus Biotechnology, São Paulo, Brazil), and comparison of the molecular weights determined the genotypes following carcinogenic potential: HR-HPV (high-risk-HPV), UR-HPV (undetermined-risk-HPV) and LR-HPV (low-risk-HPV). The following types were determined for this genotyping method: HR (−16, −18, −31, −33, −35, −39, −45, −51, −52, −53, −56, −58, −59, −66, −68, −73 and −82), undetermined risk (UR- 26) and LR (−6, −11, −30, −34, −40, −42, −43, −44, −54, −55, −61, −62, −64, −67, −69, −70, −72, −74, −81, −83, −84 and −91) (International Agency for Research on Cancer-IARC).

### Statistical analysis

Statistical analysis was performed using EPI INFO 7.0 software (CDC, Atlanta, GA, USA). All the variables were expressed as absolute and relative frequencies. The Chi-square test and a crude odds ratio (OR) with 95% confidence interval (CI) were calculated. A *p* value <0.05 was considered statistically significant.

## Results

We evaluated 52 semen samples from men subjected to semen analysis due conjugal infertility (68.4%) and 24 due post-vasectomy control (31.6%). Overall, the men had a mean of 1.2 ± 1.9 children (range 0–5), and this value was 0.5 ± 0.9 (range 0–4) for those experiencing conjugal infertility.

**Figure 1:**
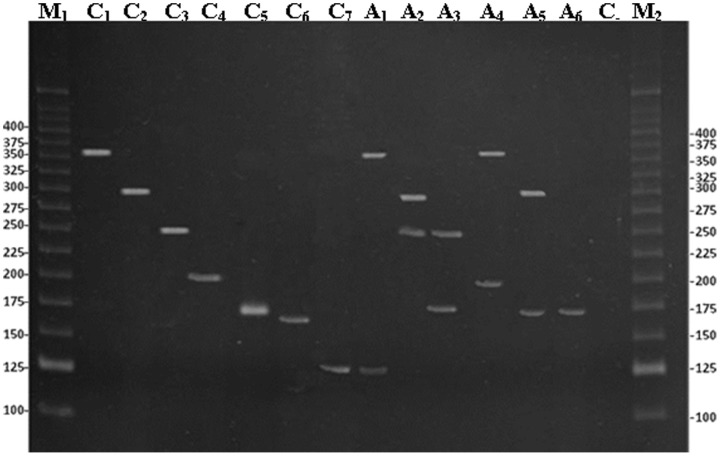
Electrophoretic analysis of the amplified fragments by using a multiplex polymerase chain reaction in 8% polyacrylamide gel stained with ethidium bromide. Lane C_1_: control of *Chlamydia trachomatis* (361 base pairs-bp); lane C_2_: control of *Treponema pallidum* (291 bp); lane C_3_: control of HSV–2 (249 bp); lane C_4_: control of *Mycoplasma genitalium* (193 bp); lane C_5_: control of *Trichomonas vaginalis* (170 bp); lane C_6_: control of *Neisseria gonorrhoeae* (162 bp); lane C_7_: control of HSV–1(123 bp); lane A_1_: positive sample of *C. trachomatis* and HSV–1 (361 and 123 bp); lane A_2_: positive sample of *T. pallidum* and HSV–2 (291 and 249 bp); lane A_3_: positive sample of *T. vaginalis* and HSV–2 (170 and 249 bp); lane A_4_: positive sample of *C. trachomatis* and *M. genitalium* (361 and 193 bp); lane A_5_: positive sample of *T. pallidum* and *T. vaginalis* (291 and 170 bp); lane A_6_: positive sample of *T. vaginalis* (170 bp); lanes M_1_ and M_2_, molecular weight marker (25 bp Invitrogen). Values on the left and right sides of the gel are in bp.

The M-PCR assay clearly distinguished and identified all seven STDs in semen samples whether alone (1 STD) or in co-infections. Final results were regarded as true positives if the sPCR was positive (gold standard). The overall agreement of M-PCR results with sPCR was 99.2%, and the validation parameters were as follows: sensitivity and negative predictive value 100%, specificity 99.7%, positive predictive value 96.4%, and accuracy 99.8%. When individually analysed the agents *C. trachomatis, M. genitalium, N. gonorrhoeae, T. pallidum*, *T. vaginalis* and HSV-2, the M-PCR showed values of 100% for all parameters. For HSV-1, the M-PCR showed sensitivity and negative predictive values of 100%, specificity of 98.2%, positive predictive value of 75.0%, and accuracy of 98.3% (Table 2). By M-PCR, 5 semen samples (6.6%) were detected with at least 2 simultaneous STD agents. Figure 1 shows the M-PCR amplification fragments of positive semen samples for different STDs by 8% polyacrylamide gel.

**Table 2:** M–PCR validation results compared with single PCR (sPCR) for seven clinically important STDs pathogens in semen, including *Chlamydia trachomatis*, *Mycoplasma genitalium*, *Neisseria gonorrhoeae*, *Treponema pallidum, Trichomonas vaginalis*, HSV–2 and HSV–1.

M–PCR parameters	General results (%)	CT (%)	MG (%)	NG (%)	TP (%)	TV (%)	HSV– 2 (%)	HSV–1 (%)
Sensitivity	100.00	100.00	100.00	100.00	100.00	100.00	100.00	100.00
Specificity	99.70	100.00	100.00	100.00	100.00	100.00	100.00	98.20
PPV	96.40	100.00	100.00	100.00	100.00	100.00	100.00	75.00
NPV	100.00	100.00	100.00	100.00	100.00	100.00	100.00	100.00
ACC	99.80	100.00	100.00	100.00	100.00	100.00	100.00	98.3

M–PCR, multiplex–polymerase chain reaction; sPCR, single polymerase chain reaction; STDs, sexually transmitted diseases; PPV, positive predictive value; NPV, negative predictive value; ACC, accuracy; CT, *C. trachomatis*; TP, *T. pallidum*; HSV–1/–2: herpes virus simplex; MG, *M. genitalium*; TV, *T. vaginalis*; NG, *N. gonorrhea*.

Considering the 7 STDs detected by M-PCR and HPV by sPCR, 1 or more STDs were detected in 42 semen samples (55.3%). The most prevalent STD detected was HPV (*n* = 29), representing 38.1% of the total samples and 69.0% of the semen positive for STDs. The second most prevalent was *T. vaginalis* (*n* = 10; 13.0% in total samples and 23.8% in semen with STD), followed by *C. trachomatis* (*n* = 6; 8.0% in total samples and 14.3% in semen with STD), HSV-1 and *T. pallidum* (*n* = 4; 5.3% in total semen samples and 9.5% of semen with STD, each), *M. genitalium* and *N. gonorrhoeae* (*n* = 3; 4.0% in total samples and 7.1% in semen with STD, each) and HSV-2 (*n* = 2; 2.6% in total samples and 4.8% in semen with STD) (Table 3). Co-infections by 2 pathogens was in 17 out of 42 infected samples (40.5%) (Table 4). Three simultaneous STD were detected in only 1 sample (2.4% of semen with STD and 1.32% of total samples).

**Table 3:** Frequency of STDs and reason for performing the semen analysis.

STD	Total *n* (%)	Conjugal infertility *n* (%)	Post– vasectomy *n* (%)	OR (IC 95%)	*P*
HPV					
(+)	29 (38.0)	20 (38.5)	9 (37.5)	1.1 (0.4–3.0)	0.78
(−)	47 (62.0)	32 (61.5)	15 (62.5)		
HR–HPV					
(+)	24 (32.0)	16 (30.8)	8 (33.3)	0.9 (0.3–2.7)	0.90
(−)	52 (68.0)	36 (69.2)	16 (66.7)		
*C. trachomatis*					
(+)	6 (8.0)	5 (9.6)	1 (4.2)	2.5 (0.3–23.6)	0.40
(−)	70 (92.0)	47 (90.4)	23 (95.8)		
*T. vaginalis*					
(+)	10 (13.0)	7 (13.5)	3 (12.5)	1.1 (0.3–4.9)	0.83
(−)	66 (87.0)	45 (86.5)	21 (87.5)		
*M. genitalium*					
(+)	3 (4.0)	2 (3.9)	1 (4.2)	0.9 (0.0–11.3)	0.90
(−)	73 (96.0)	50 (96.1)	23 (95.8)		
HSV–1/–2					
(+)	6 (8.0)	5 (9.6)	1 (4.2)	2.5 (0.3–23.6)	0.40
(−)	70 (92.0)	47 (90.4)	23 (95.8)		
*T. pallidum*					
(+)	4 (5.3)	4 (7.7)	0 (0)	–	0.10
(−)	72 (94.7)	48 (92.3)	24 (100.0)		
*N. gonorrheae*					
(+)	3 (4.0)	2 (3.9)	1 (4.2)	0.9 (0.0–11.3)	0.90
(−)	3 (96.0)	50 (96.1)	23 (95.8)		
Total	76	52	24	–	–

STD, sexually transmitted diseases; HPV, human *Papillomavirus*; HR–HPV, high–risk human *Papillomavirus*; HSV–1/–2, herpes simplex virus; (+), positive; (−), negative.

**Table 4:** Total simultaneous STDs detected by M-PCR and sPCR.

Simultaneous STDs	*n*	Total semen samples *n* = 76 (%)	Semen samples with STD *n* = 42 (%)
*C. trachomatis* + HSV–1	2	2.6	4.8
*T. pallidum* + *T. vaginalis*	1	1.3	2.4
HSV–2 + *T. vaginalis*	1	1.3	2.4
HSV–2 + *T. pallidum*	1	1.3	2.4
HPV + *T. vaginalis*	3	3.9	7.1
HPV + *C. trachomatis*	3	3.9	7.1
HPV + HSV–1	2	2.6	4.8
HPV + *M. genitalium*	2	2.6	4.8
HPV + *N. gonorrhoe*	1	1.3	2.4
HPV + *T. pallidum*	1	1.3	2.4

STDs; sexually trasnmitted disease; M-PCR, multiplex-polymerase chain reaction; sPCR, single polymerase chain reaction; HSV -1/-2, herpes vírus simplex.

In the semen of men with conjugal infertility, the most prevalent STD was HPV (*n* = 20/52; 38.5%), followed by *T. vaginalis* (n = 7/52; 13.5%), *C. trachomatis* and HSV-1/-2 (*n* = 5/52; 9.6% each). In the semen analyzed due to post-vasectomy, the most prevalent STD was also HPV (*n* = 9/24; 37.5%), followed by *T. vaginalis* (*n* = 3; 12.5%) (Table 3). Men with *C. trachomatis* or HSV (types 1 and 2) semen infections had a 2.5-fold greater risk of presenting conjugal infertility (OR = 2.5, 95% CI = 0.3–22.7), although the association between these STDs and conjugal infertility was not statistically significant (*p* = 0.4 for both).

**Figure 2:**
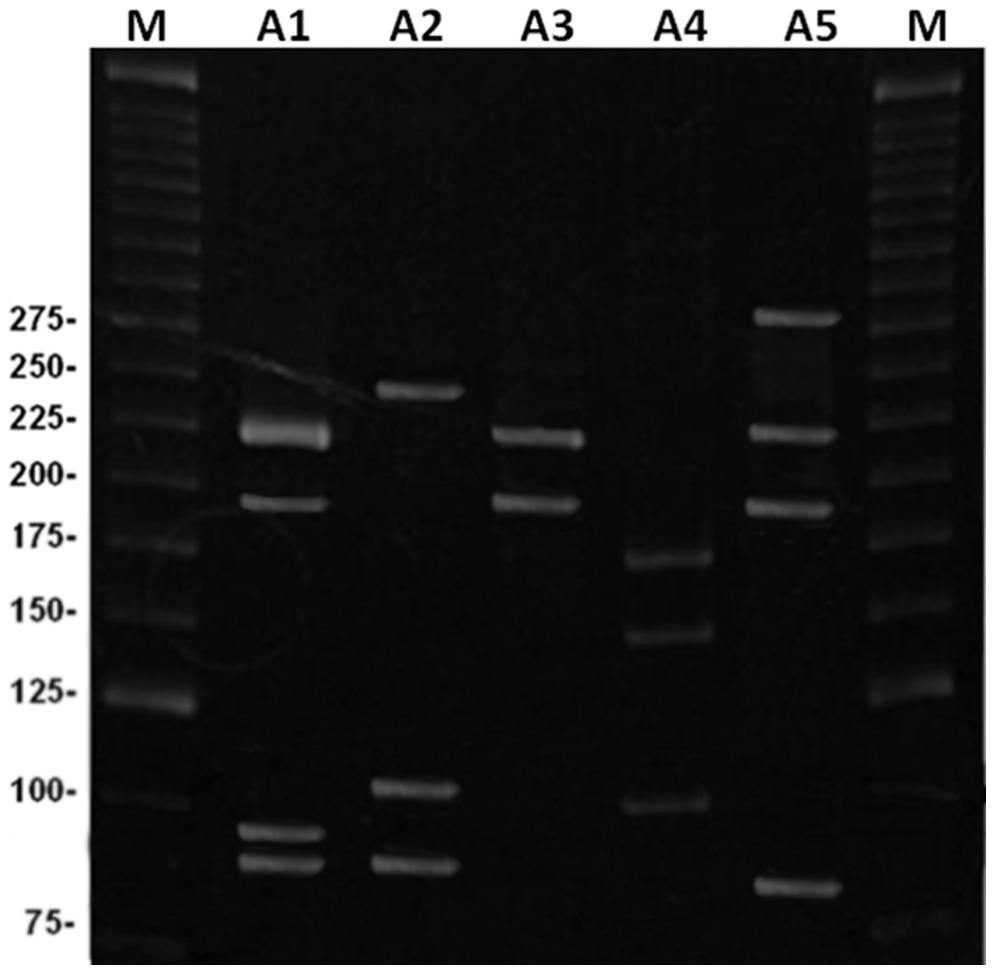
Electrophoretic analysis of the HPV genotyping in semen using PCR-RFLP with restriction enzyme *Hpy*CH4V in 8% polyacrylamide gel stained with ethidium bromide. Sample A1, genotypes −16 (High-risk, HR) and −31 (HR) in double HPV infection (216, 191, 94 and 91 base pairs-bp); A2, genotype −13 (low-risk, LR) in single HPV infection (244, 103 and 91 bp); A3, genotype −16 (HR) in single HPV infection (216 and 191 bp); A4, genotype −18 (HR) in single HPV infection (174, 144 and 100); A5, genotypes −81(LR), −66 (HR) and −16 (HR) in multiple HPV infection (284, 216, 191 and 89 bp). M, molecular weight marker (25 bp).

Among HPV positive semen (*n* = 29), 17 (58.6%) were infected by 2 or more genotypes simultaneously representing 22.5% of the total samples and 40.5% of the semen with STDs. Figure 2 shows the sPCR amplification fragments of positive semen samples for HPV by 8% polyacrylamide gel. In regarding to carcinogenic HPV groups, 24 out of 29 (82.7%) were HR-HPV. HPV-16 (HR) was the most prevalent genotype detected (*n* = 13/29; 44.8%) and was found in 17.1% of the total samples and 30.9% of the semen with STDs. HPV-82 and HPV-43 (HR) were the second most prevalent (n = 5/29; 17.2% each), followed by HPV-72 (LR, *n* = 4/29; 13.8%), −58 (HR, *n* = 3/29; 10.3%); −54 (LR) and −66 (HR) (*n* = 2/29; 6.9% each). Other HPV genotypes detected in single or simultaneous infections were HPV-13,-18,-31,-44,-51,-52,-53,-59,-62,-69 and -81 (*n* = 1/29; 3.5% each). Table 5 shows the HPV genotypes detected in simultaneous semen HPV infections. According to Santiago et al. [19], their method with a single enzyme (HpyCH4V) application is simple enough for the detection of HPV in clinical samples but cannot be used to distinguish some HPV genotypes such as HPV 11/30, 18/68, 44/55, and 61/83/84, because these genotypes yield similar RFLP patterns. Chen et al. [25] used the same enzyme (*Hpy*CH4V) for the initial RFLP and then used a second enzyme ((*Nla*III) to confirm the identification. We didn't find any of these genotypes in our analysis and the patter found by the single enzyme was very different and yielded us to well distinguish between all of them, using the 8% polyacrylamide gel as showed in Figure 2. So, as mentioned above there was not the requirement to apply another assay with the second enzyme to confirm our results.

**Table 5:** HPV genotypes distribution of 17 semem samples with two or more genotypes.

Samples	HPV genotypes		*n* (%)
	LR	HR	
1	−81	16, −66	1 (5.88)
2	_	−51, −52	1 (5.88)
3	_	16, −82	1 (5.88)
4	_	16, −31	1 (5.88)
5	−54	−82	2 (11.80)
6	−72	−53	1 (5.88)
7	−72	−58	1 (5.88)
8	−43	−82	1 (5.88)
9	−44	−82	1 (5.88)
10	−43	−58	1 (5.88)
11	−43, −72	_	1 (5.88)
12	_	−16, −59	1 (5.88)
13	_	−16, −58	1 (5.88)
14	_	−16, −56	1 (5.88)
15	−69	−16	1 (5.88)
16	−72	−16	1 (5.88)
17	−43	−16	1 (5.88)

HPV, human *Papillomavirus*; HR, high–risk human *Papillomavirus*; LR, low-risk human *Papillomavirus*.

In regarding to semen quality parameters, the following observations were made: a decrease of seminal volume in samples with 2 or 3 simultaneous STDs or *T. pallidum* alone (*p* = 0.01 and *p* = 0.02, respectively); semen with simultaneous *T. vaginalis* and HPV infections had a 10-fold greater risk of presenting teratospermia (OR = 10.55; 95% CI 0.8 to 13.3) (*p* = 0.03); a tendency toward a greater risk of semen alterations, although not statistically significant, between *T. vaginalis* and necrospermia (OR = 2.7; 95% CI 0.4 to 15.7) (*p* = 0.2), simultaneous *T. vaginalis* and HPV with oligospermia (OR = 2.8; 95% CI 0.2 to 3.3) (*p* = 0.3) and simultaneous HSV-1/-2 and HPV with teratospermia (OR = 4.6; 95% CI 0.2 to 8.2) (*p* = 0.2).

## Discussion

To our knowledge, this is the first study to detect simultaneous bacterial and viral STD pathogens in semen using M-PCR in Brazil and Latin America. The method implemented, which represented an important advance over conventional techniques, allowed the detection of seven STDs in semen, many of which are difficult to identify using standard methods. The overall agreement of the M-PCR with sPCR was elevated (99.2%), and other validation parameters, including sensitivity, specificity, positive and negative predictive value and accuracy, were also excellent (ranging from 99.2% to 100%). Considering the agents individually, the M-PCR also showed excellent values for all the parameters and detected 5 semen samples (6.6%) with co-infections.

The M-PCR assay simplifies workflow, allowing for its use in routine diagnostic laboratories with basic molecular facilities [20]–[24]. Thus, this M-PCR assay has great potential to be applied in screening programs for semen pathogens in infertility and STD clinics and in sperm banks. Further investigations applying M-PCR in different populations and clinical situations may help elucidate the impact of STD pathogens on male infertility. All encompassed 7 STDs in addition to HPV were analyzed by M-PCR and sPCR. The overall prevalence of STDs in semen from asymptomatic men was determined to mount to 42 semen samples out of 52 (55.3%) and showed 1 or more pathogens.

HPV was the most prevalent STD pathogen in semen (*n* = 29; 38.1% of total semen and 69.0% of semen with STDs). Surprisingly, in 58.6% of semen samples 2 or more genotypes were detected simultaneously (22.5% of total semen and 40.5% of positive semen for STDs) and HR-HPV represented 82.7% of all the HPV genotypes detected. The most prevalent HPV genotype was HPV-16 (*n* = 13/29, 44.8%; *n* = 13/76, 17.1%), followed by HPV-82 and HPV-43, both of which are HR-HPV (*n* = 5/29; 17.2% each). The current vaccine protects against HR HPV-16 and -18, but in our study HPV-18 genotype is not the second most frequent HR but HPV-82 and -43, which could have implications for the effectiveness of the vaccine Brazilian men.

A recent study found HPV at a much lower prevalence (10%), but the population selected for this research included only asymptomatic sexually active young men [25]. HPV infection is well-characterized in women. However, little attention has been given to the transmission of HPV through semen [26], [27] because the virus is primarily transmitted through direct epithelial contact [28]. Normally, HPV infection in men is considered temporary, but little is known about the incubation time and possible viral manifestations [29].

The second most prevalent STD was *T. vaginalis* (*n* = 10; 13.0% in total semen samples and 23.8% in semen with STDs). Knowledge of *T. vaginalis* in men, including in semen, is limited mainly due to difficulties associated with diagnosis including poor sensitivity of available methods [30], [31]. Estimates of the prevalence of trichomoniasis range from 6–12% of asymptomatic men to 20% of men with urethritis [32]. A study in females with trichomoniasis analyzed the urine and semen of their sexual partners and found that 72% of men were also positive for *T. vaginalis* despite the fact that the majority were asymptomatic [31].

*C. trachomatis* was the third most common STD (*n* = 6; 8.0% in total semen samples and 14.3% semen with STD). The number of men infected by this pathoges was similar to another study that showed that although up to 13.3% of young men carry genital *C. trachomatis* infection, only half of these will present with any symptom and even fewer are likely to pursue treatment [33]. Thus, it has been suggested that undiagnosed *C. trachomatis* infection in either partner could potentially contribute to unexplained infertility [23]. This bacterium can be located in any part of the male reproductive tract, including sexual glands, such as the prostate and seminal vesicles [34]. *C. trachomatis* infections of male sexual glands may cause severe complications that can threaten male fertility [23]. Urethritis is the most common clinical presentation of *C. trachomatis* infection observed in men [35], but it is normally an acute episode of chronic and silent genitourinary infection [36]–[38]. Evidence also suggests that upper genital tract infections in young men, including epididymitis, are attributable to *C. trachomatis* [37]–[39]. Epididymitis is thought to be important because fertility can be affected by inflammation and obstruction, especially when both testes are affected [35].

Other STD pathogens were detected in lower abundances as follows: HSV-1 and *T. pallidum* (*n* = 4/76; 5.3% each), *M. genitalium* and *N. gonorrhoeae* (*n* = 3/76; 4.0% each) and HSV-2 (*n* = 2/76). No previous studies were found assessing the prevalence of *T. pallidum* in semen. Although a direct toxic effect of syphilis on male fertility has not been reported in the literature, it is known that complications of syphilis can affect fertility. Syphilitic epididymitis can cause obstruction of the epididymis. Chronic obliterative endarteritis and interstitial inflammation can occur in congenital or tertiary syphilis and lead to small, fibrotic testes [40]. Gummatous lesions cause destruction of local tissue and, when occurring in the testicles, may have an impact on testicular function and fertility [41].

Among men presenting persistent or recurrent urethritis, 19% to 41% are infected with *M. genitalium* [42]. This bacterium is a probable cause of non-gonococcal urethritis [43], [44] and is also associated with prostatitis [24], [45]–[49]. The detection of genital mycoplasmas only in semen may indicate that these organisms are harbored in the epididymis or seminal vesicles. The influence of mycoplasmas on semenology may come from their ability to attach to spermatozoa and directly affect cellular interactions, influencing vitality, motility, morphology, cellular integrity, molecular structure and the development of protective immunity to genital infection by the host or other host factors [24].

*N. gonorrhoeae* infection in men can lead to genitourinary tract inflammation (e.g., urethritis and epididymitis), obstruction and infertility [50]–[52]. Gonococcus is transmitted more efficiently from an infected male to a female (50% to 73% probability, independent of the number of exposures) [50], [53], [54]. Because *N. gonorrhoeae* is mostly symptomatic in males, there have been very few studies regarding the relationship between this infection and male infertility. Surprisingly, we detected HSV-2 in semen less frequently than HSV-1. Most commonly, HSV-1 causes oral and genital sores, but HSV-2 is the most common cause of genital herpes. Generally, direct or indirect contact with herpetic lesions is infectious, but HSV-1 and HSV-2 have been detected in semen [22], [55] and in sperm, and HSV-2 has been transmitted through donor insemination [56], [57]. DNA of HSV-1 and HSV-2 has been detected in the semen from 2-50% of men with no significant difference between fertile and infertile subjects [22], [56], [58]. Evidence indicates that HSV infections contribute to male factor infertility either by directly invading male genital tract cells or by indirectly causing local immune responses that can negatively affect reproduction [59]. More specifically, HSV infection has been correlated to reduced sperm count, progressive motility, increased apoptosis and low sperm concentration [60], [61].

Other viruses can be highly frequent in semen such as cytomegalovirus (CMV) [8], [58] which is well known a STD pathogen [17]. CMV could be one of the most frequent pathogens in semen. However it was not investigated in our study, since our aim was adapt the M-PCR assay for seven agents as used by Souza et al. [14] in cervical samples to semen samples and we succeed. Thus, a new M-PCR assay allowing the detection of other STD agents would be interesting and a promising research to be standardized.

Despite being a secondary goal of our study some associations were observed between STDs and changes in semen parameters such as: decreased seminal volume in samples with 2 or 3 simultaneous STDs or *T. pallidum* alone; semen with simultaneous *T. vaginalis* and HPV infections with a 10-fold greater risk of teratospermia. Correlations were also observed in: *T. vaginalis* with necrospermia; *T. vaginalis* and HPV co-infections with oligospermia; HSV-1/-2 and HPV co-infections with teratospermia. These associations confirmed the involvement of STD pathogens with male infertility as described in few previous studies [7]–[9].
