# Correction: Sensitive Simultaneous Detection of Seven Sexually Transmitted Agents in Semen by Multiplex-PCR and of HPV by Single PCR

**DOI:** 10.1371/journal.pone.0112864

**Published:** 2014-11-07

**Authors:** The PLOS ONE Staff

In the Funding section, a grant number from the funder Coordenação de Aperfeiçoamento de Pessoal de Nível superior (CAPES) is missing. The second grant number is PVE A109/2013.
